# Effects of Dance Therapy on Balance and Risk of Falls in Older Persons

**DOI:** 10.1093/geroni/igaa057.756

**Published:** 2020-12-16

**Authors:** Esha Chakravarty, Indrani Chakravarty, Ipsito Chakravarty, Prasenjit Bhattacharjee

**Affiliations:** 1 Calcutta Metropolitan Institute of Gerontology, Mitcham, England, United Kingdom; 2 Calcutta Metropolitan Institute of Gerontology, Kolkata, United States; 3 Calcutta Metropolitan Institute of Gerontology, Kolkata, West Bengal, India

## Abstract

Loss of balance and risk of falls is a major problem in older persons. Literature shows increasing use of yoga practices and dance therapy across Indian oldage homes and day care centres to improve balance and reduce risk of falls in older persons. Aim of this study is to evaluate the effects of dance therapy with focus on therapeutic movements derived from Indian classical dances on balance and risk of falls in older adults of Day Care Centres in Calcutta Metropolitan Institute of Gerontology, under Ministry of Social Justice and Empowerment, Govt. of India. Total of 24 older adults across 2 day care centres participated in the study attending dance therapy sessions for 3 months. All of them self reported problems of balance and repeated falls alongwith difficulties in performing Activities of Daily Living. Twenty one of them were females and 3 males. The mean age of the participants was 75.5 years. Limits of Stabililty (LOS) was used to measure balance and pre tests and post tests were performed. Results showed that the Limits of Stability were significantly higher (17.5%) in older persons after participating in the dance therapy sessions. This study supports that dance therapy using movements derived from Indian classical dance forms can support older persons to function with reduced risk of falls, improved balance, safely carry out mobility tasks and perform better Activities of Daily Living . Further studies can show how dance therapy can facilitate healthy ageing and influence State policies on healthy ageing.

